# P-455. Post-Transplantation Infection and Rejection Outcomes in Kidney Transplant Recipients Living with Human Immunodeficiency Virus (HIV): A Single-Center Experience Over a Decade of Evolving Practices

**DOI:** 10.1093/ofid/ofae631.655

**Published:** 2025-01-29

**Authors:** Meghna Nagam, Anne Huml, Emilio Poggio, Anita R Modi

**Affiliations:** Cleveland Clinic, Cleveland, Ohio; Cleveland Clinic Foundation, Cleveland, Ohio; Cleveland Clinic Foundation, Cleveland, Ohio; Cleveland Clinic Foundation, Cleveland, Ohio

## Abstract

**Background:**

The development of integrase inhibitor and injectable antiretroviral therapy (ART), use of lymphocyte-depleting induction, and implementation of the HIV Organ Policy Equity (HOPE) Act have revolutionized kidney transplant eligibility, peri-transplantation management, and post-transplantation outcomes for people living with HIV and advanced kidney disease in the last decade. We describe our experience with HIV-positive recipients over this important period.

**Methods:**

We conducted a retrospective chart review of all adults living with HIV and advanced kidney disease who underwent kidney transplantation at our center between 2013-2024. Data was collected on demographics, ART and induction regimens, and post-transplantation outcomes: patient/allograft survival, infections, and rejection episodes within the first year.

**Results:**

Sixteen patients (14 Male, 13 Black, Mean Age 51y) underwent 17 kidney transplantations. Ten transplantations occurred from 2013-2022; seven occurred within 18 months of our center’s implementation of the HOPE Act. HIV-associated nephropathy accounted for all six transplantations from 2013-2017; hypertension and diabetes mellitus accounted for most transplantations in later years. Four patients transplanted prior to 2017 received basiliximab, of whom one experienced rejection within a year; 13 subsequent transplantations were induced with anti-thymocyte globulin (ATG), with three complicated by rejection. Rejection incidence was comparable between recipients of 3 mg/kg and 4.5 mg/kg ATG. Two patients suffered graft loss (rejection, BK nephropathy). Eleven patients had infections within a year, predominantly urinary tract infections and cytomegalovirus viremia. No opportunistic infections were identified. Three out of the four patient deaths in this cohort were due to complications of coronavirus disease 2019.

**Conclusion:**

Recent advancements in HIV care and solid organ transplantation practices converged to facilitate pre-transplantation optimization, increase opportunities for transplantation, and improve post-transplantation outcomes for people living with HIV and advanced kidney disease. When used with integrase inhibitor-based therapy, ATG can prevent rejection without precipitating opportunistic infections.

**Disclosures:**

**All Authors**: No reported disclosures

